# 

**Published:** 1883-05

**Authors:** 


					﻿Vol. III.
MAY, 1883.
NO. 5.
THE
HOMEOPATHIC pHY^lClAfl,
A MONTHLY JOURNAL OF MEDICAL SCIENCE.
“ Magna est verilas, et prtBvalebii.”
ZE. 0". ZLHjEj, im:. J?.,
EDITOR.
CONTENTS :
(See inside of Cover.)
PHILADELPHIA:
No. 2109 CHESTNUT STREET.
3S5T 333 "W "3T O ZR. 3C :
A. L. CHATTERTON, PUBLISHING COMPANY, Publisher’s Agents.
T. O OST 3D O 3ST :
ALFRED HEATH & CO., Sole Agents for Great Britain,
114 Ebury Street, Eaton Square.
Yearly Subscription, $2.50, invariably in advance.
Entered at the Philadelphia Post-Office as Second Class Mail Matter.
				

